# Medical News and Miscellany

**Published:** 1895-07

**Authors:** 


### Medical News and Miscellany.

---

Dr. Thomas R. Pettway, formerly of Missisippi, has located in Austin.

---

Dr. A. Jay Sibley, of Austin, Texas, has moved to Carl, Travis county, Texas.

---

Dr. J. F. Wilson, of Bertram, Texas, died on May 27th, after an illness of nearly five years.

---

Dr. Isaac J. Jones, of this city, was recently elected by the Board of Managers physician to the Confederate home.

---

**In Sunstroke.**—An exchange recommends jaborandi in cases of sunstroke where suppression of perspiration, increased heat and high temperature exist.

---

Drs. Goodall and Joseph S. Wooten, sons of Dr. T. D. Wooten, of Austin, have returned from New York where they graduated from the College of Physicians and Surgeons.

---

**A New Journal.**—*The Monthly Retrospect of Medicine and Pharmacy* is a new publication just received at this office. Its editor is Dr. E. H. Gingrich, of Philadelphia. Price, $1.00.

---

**Roquefort Cheese.**—There is bad news for the lovers of this highly-flavored delicacy. There is a wide-spread outbreak of disease among the 800,000 ewes of the Aveyron region, from the milk of which the cheese is made to the value of $4,000,000 annually.—*Notes on New Phar. Prod.*

---

**Struck Twice.**—The lightning in and around Kansas City has always done queer things. Dr. Block, of that city, reports a case of extensive lupus in a female due to a stroke of lightning. This patient was struck twice before the lupus manifested itself. Perhaps another "bolt from the blue" would cure the disease, after *similia similibus curantur.* See?

---

**Aseptic Peritonitis.**—A recent issue of a foreign journal (says the *Boston Medical and Surgical Journal*) reports two cases of acute aseptic peritonitis, work of Hartmann and V. Morax. In one of the cases, the spleen was found with a double twisted pedicle, changed into a reddish-brown mass, and was removed. In the second case, an ovarian cyst was found, with a twisted pedicle. There was fibrin and purulent material mixed with the peritoneal fluid in both cases, but a careful bacteriological examination showed that this material was sterile. Both cases recovered without special peritoneal toilet.

---

**An Outrage in a Civilized Portion of this Country.**—A dispatch on the 6th instant tells a horrible story of four negroes who had been isolated in a swamp, near Memphis, Tennessee, and left to die of small-pox. Dr. F. S. Raymond, superintendent of the county board of health, made a visit to this section of the country. and upon his return reported that in a tent, pitched on stilts in mud and water a foot deep, he found the corpse of a negro man who died Thursday, and by his side another victim in the last stages of the disease. In another tent was a man at the point of death, and a woman almost exhausted from the strain of nursing him. The dead negro was buried, and the Mississippi authorities were telegraphed for permission to remove the others, but this, Dr. Raymond says, was refused, and they were left to die.

---

**A Narrow Escape.**

---

AUSTIN, TEXAS, June 10, 1895.

*Editors Texas Medical Journal:*

As the files of such a medical journal as the Red-back is the proper repository for everything worthy of presentation, I offer you the following: On April 12th, J. D., an inmate of the

"Texas Confederate Home," under the impression that they were "some of the new fangled cathartic pills," swallowed four tablets of P. D. & Co.'s bichloride mercury tablets of 1.41 gr. each, a total dose of 5.64 grs. I was on the premises and was summoned immediately. Found him suffering from intense collapse, though he had not swallowed the drug more than ten minutes. Having no stomach pump at hand, I gave him 1-5 gr. apomorphia, hypodermically, and the whites of three eggs. He began vomiting profusely in a few minutes and continued to do so, until the stomach was thoroughly evacuated, and vomiting of pure bile occurred. I continued the egg albumen and other demulcent drinks, and aside from a profuse diarrhœa and mild ptyalism, no further symptoms of poisoning occurred. While many cases have recovered from doses of ten to twenty grains, and while in other cases two grains have proven fatal, it should be remembered that these doses consisted of the crude salt and not accompanied by any solvent. In this case, however (tablets), the salt is finely triturated and is perfectly soluble, and contains the citric acid which is necessary to prevent the coagulation of the albuminous tissues which limits its corrosive action and its absorption. I. J. JONES, M. D.

